# Outcome in very preterm infants: a population-based study from a regional center in Austria

**DOI:** 10.3389/fped.2024.1336469

**Published:** 2024-02-02

**Authors:** Karin Konzett, David Riedl, Anya Blassnig-Ezeh, Stefanie Gang, Burkhard Simma

**Affiliations:** ^1^ Department of Pediatrics, Academic Teaching Hospital, Landeskrankenhaus Feldkirch, Feldkirch, Austria; ^2^ Department of Psychiatry and Psychotherapy, Medical University of Innsbruck, Innsbruck, Austria

**Keywords:** morbidities, mortality, regional outcome, quality assessment, preterm infants

## Abstract

**Aim:**

To determine short-term morbidity and mortality rates in the first state-wide Austrian neonatal cohort and comparison to (inter)national data.

**Methods:**

Observational, population-based cohort study, analyzing data of preterm infants (<32 + 0 weeks of gestation) born between 2007 and 2020 (*n* = 501) in an Austrian state who were admitted to the neonatal intensive care unit. Outcome criteria were mortality, neonatal morbidities: bronchopulmonary dysplasia (BPD), *severe* necrotizing enterocolitis (NEC), severe intraventricular hemorrhage (IVH grades III–IV), severe retinopathy of prematurity (ROP grades III–V) and survival-free of major complications.

**Results:**

Overall survival rate was 95%, survival free of major complications was 79%. Prevalence for BPD was 11.2%, surgical NEC 4.0%, severe IVH 4.6%, and for severe ROP 2.6%, respectively. In the extremely low gestational age neonates (ELGAN) born <28 weeks of gestation (*n* = 158), survival was 88% and survival free of major complications 58.8%. Over time, mortality decreased significantly, predominantly driven by the improvement of infants born <28 week of gestation and survival free of major complications improved.

**Conclusions:**

This study demonstrates a very low mortality rate that decreases over time. Short-term morbidities and survival free of major complications do not differ from (inter)national data in a similar group of very preterm infants. Standard operating procedures, simulation trainings and accordance to international trials may improve patient care and surpass center case loads.

## Highlights

• First state-wide Austrian study including 501 very preterm infants shows a low mortality, low morbidity and encouraging survival free of major complication rates
• Parameters do not differ from (inter)national data and improve over time, especially in extremely low birth gestational age neonates
• Good results can be reached in a medium-provider volume neonatal intensive care unit with a well organized network

## Introduction

Preterm infants with very and extremely low birth weights are at a high risk for morbidity and even mortality. Cognitive and motor development as well as behavioral outcomes are determining factors for lifetime well-being. Changes in pre- and postnatal decision-making, modified and improved strategies in delivery room management, high-end neonatal and intensive care medicine, and progressing post-discharge management lead to improved outcome (1, 2). In Austria, the current border of viability decreased and now lies at 23 weeks of gestational age (GA), with an individual recommendation for the postnatal management of infants born at the border of viability of 23 + 0–23 + 6 GA (1, 3)_._

In Austria, the proportion of very preterm infants born before 32 weeks of GA is approximately 1% of all live births (2). Since 2007, we have prospectively entered anonymized data on all very preterm infants in a local register. National guidance for setting up standardized neonatal data was established in 2012 by a working group of neonatologists and pediatric intensivists (4, 5), and data were stored in a national quality assessment program named “Österreichisches Frühgeborenen Outcome Register (ÖFGOR)”. Data submission to this register is aimed for all institutions caring for very low birth weight infants, but only neurodevelopmental outcome examination is mandatory by law (3). Recently, national data for this population were published by the Austrian Preterm Outcome Study Group in 2019 (4).

## Aim

This study reports the first state-wide outcome rates for survival, short-term morbidities, and survival free of major complications in very preterm infants over a 14-year period in a regional center of an Austrian neonatal intensive care unit (NICU). We evaluate the changes over time, discuss the results in comparison with national and international data and offer tools to surpass smaller center caseloads.

## Material and methods

In this population-based registry study, we analysed data on all very preterm infants between 23 + 0 and 31 + 6 weeks of GA, delivered from January 1, 2007, to December 31, 2020, in the state of Vorarlberg, Austria, who were admitted to the NICU. We included all infants with congenital malformations and excluded all stillbirths and patients who died in the delivery room. In addition, we calculated data of ELGAN, who were born before 28 + 0 weeks of GA (6)_._ We divided the period into two equal time frames (P1 from 2007 to 2013 and P2 from 2014 to 2020). The study reflects information from three units, two neonatal intermediate units *(NIMCU),* and one combined NICU. All preterm infants (*n* = 501, mean 36 very preterm infants per year) are either born at Feldkirch Academic Teaching Hospital, or are transferred to the NICU there within their first day of life. Our concept of regionalization provides and supports antenatal transport. To our knowledge, only a small number of very preterm infants (*n* = 49, 8.9%) were delivered in others regions of Austria for no medical reasons (7). The infrastructural requirements, which we fulfill, are defined in the Austrian Structural Health Care Plan (Österreichischer Strukturplan Gesundheit 2017) (3) and include the coverage of essential staff (neonatologist, anesthesiologist and nursing staff) over 24/7 days. Since 2007, neonatal data have been prospectively collected, anonymized, and stored electronically in an internal register. A secure interface protected confidentiality and privacy of data. A local study coordinator (K.K.), as the one who has access to the register by a secure password, was responsible for data collection and quality control. Sociodemographic and clinical data were extracted from the register and included the patients' GA, birth weight, sex, administration of antenatal steroids, multiple births, mode of delivery, and APGAR scores (2, 5).

### Neonatal data

The primary outcome measure was overall survival, which was defined as the number of infants admitted who were discharged alive. The secondary outcome measures were the four major adverse short-term morbidities: bronchopulmonary dysplasia (BPD), necrotizing enterocolitis (NEC), intraventricular haemorrhage (IVH), retinopathy of prematurity (ROP), and survival-free of major complications.

Overall mortality included all deaths that occurred during the first admission to our NICU until discharge from the hospital. All stillbirths and patients who died in the delivery room were excluded, and infants with congenital malformations were included in our calculations.

In accordance with the definition given by Jobe & Bancalari (8), BPD was defined as the requirement for supplemental oxygen at 36 weeks of postconceptional age or discharge to home, whichever came first. BPD was not graded according to severity (8). As we took part in the NIPPV (9) and SAIL (10) trials the definition of BPD changed in the second period of the study to respiratory support regardless of FiO_2_ to maintain oxygen saturation ≥90% and to perform an oxygen reduction test (11–13). A completed course of antenatal steroids was considered as two doses with a 24-h interval, with the last dose administered more than 24 h before birth. NEC was defined according to Bell's criteria (14), where severe NEC requires surgical intervention. IVH was classified according to Papile et al. (15) and severe IVH was graded as IVH ≥grade III. ROP was graded in conformity with the international classification, and grades III–V were classified as severe ROP (16). Adverse short-term outcomes were defined as the development of any of the following adverse morbidities: BPD, severe NEC, severe IVH (grades III and IV), or severe ROP (grades III–V). The percentage of the cohort who survived without any of the four major short-term morbidities was summed up as survival free of major complications.

### Ethics

The nationwide registry was approved by the Ethics Committee of the Medical University of Vienna (EK 1828/2019). The study was approved by the Ethics Committee of the State of Vorarlberg (EK-2-7/2020), conducted in accordance with the Declaration of Helsinki and followed the Strengthening the Reporting of Observational Studies in Epidemiology (STROBE) reporting guidelines for cohort studies. The authors disclose any potential conflicts of interest.

### Statistical analysis

Data analyses were performed using SPSS software, version 21.0 for Windows (IBM; Armonk, New York, USA). Descriptive statistics are provided as percentages unless otherwise stated. Group differences were analysed using the chi-square test (*χ*2). Statistical significance was set at *p* < 0.05.

## Results

In total, 501 infants (250 in P1, 251 in P2) with a mean birth weight of 1190 (+/−354) grams and a mean GA of 28.5 (+/−2.0) weeks were included. Delivery room deaths were excluded from the study. There were no missing data on sex or mortality. For mode of delivery and single vs. multiple births, missing data were low, for BPD, NEC, IVH, and ROP, with a maximum of 1.2% for NEC. For antenatal steroids, missing data were 8%.

Neonatal characteristics were summarized using GA (Table 1). Male sex (53.9%), mode of delivery (C-section 92.8%), multiple births (34.5%), mean birth weight, and mean GA remained similar over time (Table 2).

**Table 1:** Characteristics of all included infants listed for gestational age.

Gestational age (weeks)														
Characteristics *N* (%)	Total	23–27	28–31	*p*-value	23	24	25	26	27	28	29	30	31	*p*-value
Admissions	501	158 (31.5%)	343 (68.5%)	<.001	5	23	30	41	59	62	75	86	120	
Average birth weight (SD)	1,190 (354.4)g	854.9 (228.1)	1,344.8 (289.5)	<.001	531.2 (109.9)	640.3 (91.1)	742.3 (172.9)	872.7 (198.8)	1,010.8 (189.4)	1,127.1 (188.4)	1,200.0 (212.4)	1,332.5 (235.7)	1,190.3 (354.4)	<.001
Mortality	25 (5.0%)	19 (12%)	6 (1.7%)	<.001	1 (20.0%)	6 (26.1%)	8 (26.7%)	2 (4.9%)	2 (3.4%)	1 (1.6%)	3 (4.0%)	1 (1.2%)	1 (0.8%)	<.001
Male sex	270 (53.9%)	90 (57.0%)	180 (52.5%)	.35	3 (60.0%)	12 (52.2%)	18 (60.0%)	24 (58.5%)	33 (55.9%)	38 (61.3%)	39 (52.0%)	44 (51.2%)	59 (49.2%)	.87
Antenatal steroids	422 (84.2%)	138 (87.3%)	284 (82.8%)	.37	4 (80.0%)	22 (95.7%)	24 (80.0%)	37 (90.2%)	51 (86.4%)	54 (87.1%)	66 (88.0%)	71 (82.6%)	93 (77.5%)	
Incomplete course	75 (15.0%)	31 (19.6%)	44 (12.8%)	.18	3 (60.0%)	5 (21.7%)	8 (26.7%)	7 (17.1%)	8 (13.6%)	6 (9.7%)	14 (18.7%)	9 (10.5%)	15 (12.5%)	.06
Complete course	347 (69.3%)	107 (67.7%)	240 (70.0%)	?	1 (20.0%)	17 (73.9%)	16 (53.3%)	30 (73.2%)	43 (72.9%)	48 (77.4%)	52 (69.3%)	62 (72.1%)	78 (65.0%)	
Multiple births	173 (34.5%)	40 (25.3%)	133 (38.8%)	.004	1 (20.0%)	5 (21.7%)	9 (30.0%)	4 (9.8%)	21 (36.2%)	17 (27.4%)	30 (40.0%)	35 (40.7%)	51 (42.5%)	.008
PROM	173 (34.5%)	57 (36.1%)	116 (33.8%)	.89	3 (60.0%)	6 (26.1%)	5 (16.7%)	18 (43.9%)	25 (42.4%)	23 (37.1%)	27 (36.0%)	25 (29.1%)	41 (34.2%)	.31
C-section	465 (92.8%)	145 (91.8%)	320 (93.3%)	.20	4 (80.0%)	21 (91.3%)	27 (90.0%)	38 (92.7%)	55 (93.2%)	59 (95.2%)	71 (94.7%)	81 (94.2%)	109 (90.8%)	.70
Place of birth				<.001										.018
Inborn	376 (75.0%)	136 (86.1%)	240 (70.0%)		4 (80.0%)	23 (100.0%)	25 (83.3%)	37 (90.2%)	47 (79.7%)	49 (79.0%)	51 (68.0%)	54 (62.8%)	86 (71.7%)	
Outborn	120 (24.0%)	20 (12.7%)	100 (29.2%)		1 (20.0%)	0 (0.0%)	5 (16.7%)	3 (7.3%)	11 (18.6%)	13 (21.0%)	23 (30.7%)	32 (37.2%)	32 (26.7%)	
Missing	5 (1.0%)	2 (1.3%)	3 (0.9%)		0 (0.0%)	0 (0.0%)	0 (0.0%)	1 (2.4%)	1 (1.7%)	0 (0.0%)	1 (1.3%)	0 (0.0%)	2 (1.7%)	
SGA	48 (9.6%)	19 (12.0%)	29 (8.5%)	.21	3 (60.0%)	3 (13.0%)	5 (16.7%)	5 (12.2%)	3 (5.1%)	3 (4.8%)	7 (9.3%)	11 (12.8%)	8 (6.7%)	.004

**Table 2:** Characteristics of all included infants listed over time.

Characteristics *N* (%)	Total	P1 2007–2013	P2 2014–2020	*p*-value
Admissions	501	250	251	.94
Average birth weight (SD)	1190.3 (354.4)	1191.3 (339.4)	1189.2 (369.5)	.95
Average GA (SD)	28.5 (2.0)	28.6 (2.0)	28.4 (2.2)	.32
Mortality	25 (5.0%)	16 (6.4%)	9 (3.6%)	.15
Male sex	270 (53.9%)	134 (53.6%)	136 (54.2%)	.90
Extremly preterm infants	158 (31.5%)	74 (29.6%)	84 (33.5%)	.35
Antenatal steroids	422 (84.2%)	217 (86.8%)	205 (81.7%)	.74
incomplete course	75 (15.0%)	38 (15.2%)	37 (14.7%)	.94
complete course	347 (69.3%)	179 (71.6%)	168 (66.9%)	
Multiple births	173 (34.5%)	96 (38.4%)	77 (30.7%)	.07
PROM	173 (34.5%)	72 (28.8%)	101 (40.2%)	.002
C-section	465 (92.8%)	231 (92.4%)	234 (93.2%)	.70

### Mortality

**Figure 1:**
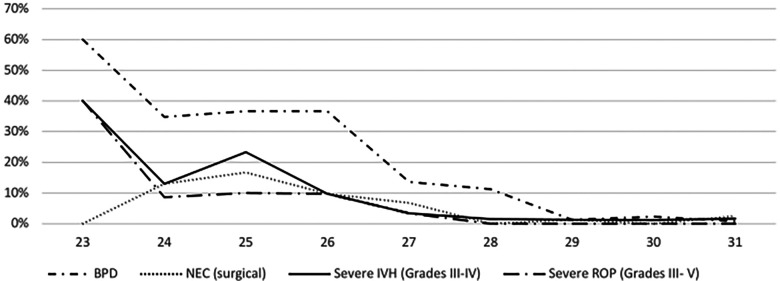
Incidences of short term morbidities (BPD, surgical NEC, severe IVH, severe ROP).

Overall, 25 very-preterm infants died (5.0%). For *ELGAN*, the mortality rate was 12%, and for those >28 weeks of GA, it was 1.7% (Table 2). The death rates in infants at 23, 24, 25, 26, and 27 weeks of GA were 20%, 26.1%, 26.7%, 4.9%, and 3.4%, respectively. The corresponding death rates for infants at 28, 29, 30, and 31 weeks of GA were 1.6%, 4.0%, 1.2%, and 0.8%, respectively (Table 1, Figure 1). Mortality decreased from 6.4% in P1 to 3.6% in P2 (*p* = .14) (Table 2).

### Short-term morbidities

**Figure 2:**
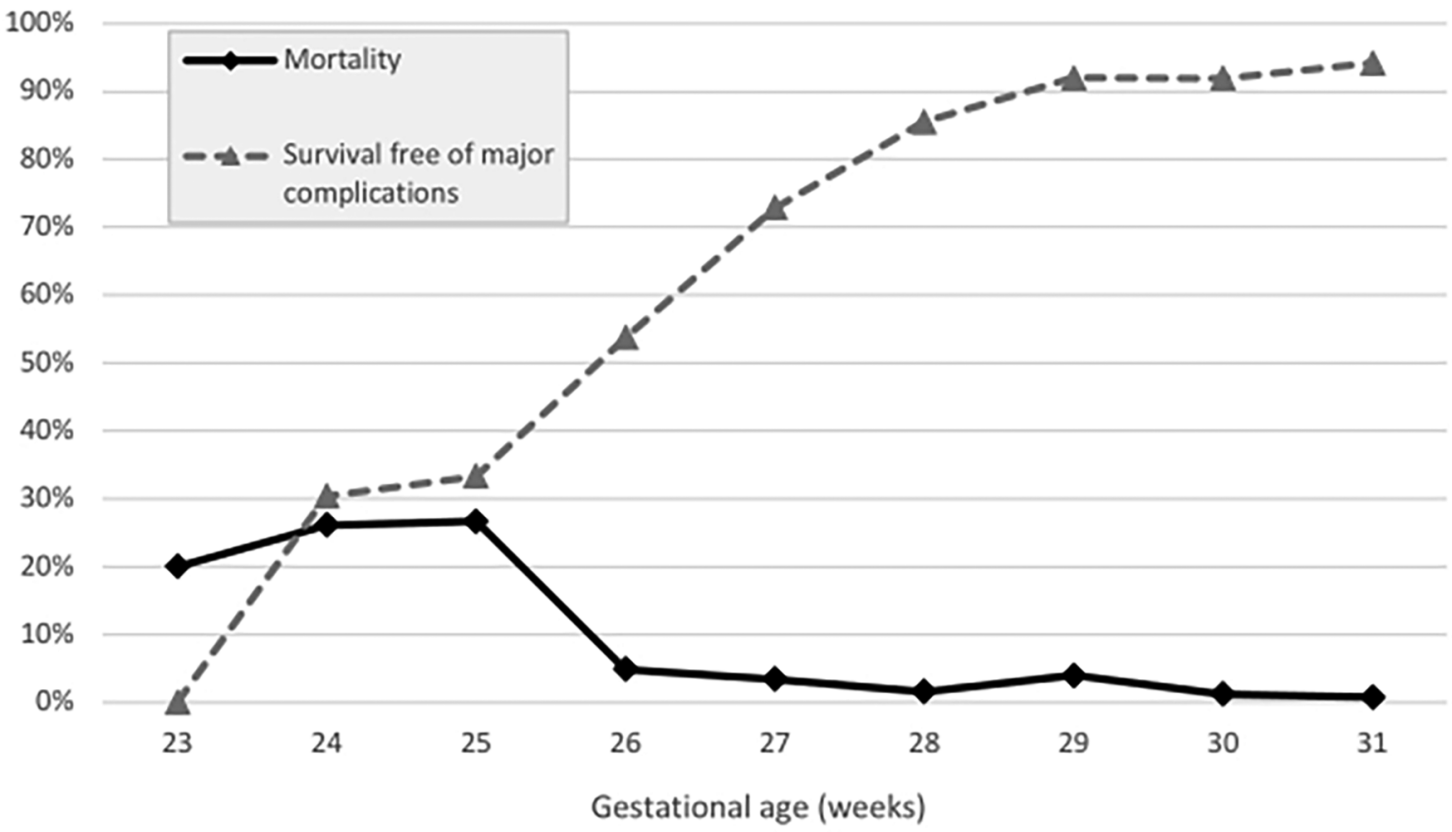
Mortality vs. survival free of major complications (in percent) stratified across gestational age groups (weeks).

Overall, the incidence rates were 11.2% for BPD, 4.0% for surgical NEC, 4.6% for severe IVH, and 2.6% for severe ROP. BPD rates significantly increased from P1 to P2 (7.2% to 15.1%, *p* = .04), whereas other morbidities showed non-significant changes from P1 (2.4% for NEC, 4.8% for IVH, 1.6% for ROP) to P2 (5.6% for NEC, 4.4% for IVH, 3.6% for ROP). For detailed information, see Figure 2.

### Adverse short-term outcome

The risk to be discharged with one or more of the predefined morbidities was 10.6% for one, 3.3% for two and 0.8% for three morbidities. No infants had all of the four morbidities at discharge. In ELGAN, the risk for at least one short-term morbidity ranged from 69.6% with 24 weeks of GA to 14.5% in those with 28 weeks and 5.8% in those with 31 weeks of GA.

### Survival free of major complications

Overall, the survival free of major complications rate was 79.0%. It ranged from 30.4% in infants with 24 weeks of GA to 94.2% in those with 31 weeks of GA (Figure 1), and showed no significant difference over time (82.4% for P1 to 75.7% for P2, *p* = .15).

#### Extremely low gestational age neonates (ELGAN)

In this group of infants born below 28 + 0 weeks of GA, mean birth weight was 854.9 (+/−228.1) grams. The mortality rate was 12.0% and decreased significantly from 19% in P1 to 6.0% in P2 (*p* = .012) (Table 3). The incidence of short-term morbidities was 28.5% for BPD, with a significant increase over time (18.9% in P1 vs. 36.9% in P2, *p* = .025), 10.1% for surgical NEC, 11.4% for severe IVH, and 8.2% for severe ROP, with no changes over time. The overall survival free of major complications in this group was 51.9%, with no significant change over time (Table 3).

**Table 3:** Characteristics of all extremely low gestational age neonates.

Characteristics *N* (%)	Total	P1 2007–2013	P2 2014–2020	*p*-value
Admissions	158 (31.5%)	74 (46.8%)	84 (53.1%)	.96
Average birth weight (SD)	854.9 (228.1)	861.0 (201.3)	849.5 (250.5)	.75
Mortality	19 (12.0%)	14 (18.9%)	5 (6.0%)	<.001
*GA: week 23–25*	*15* (*78.9%)*	*12* (*85.7%)*	*3* (*60.0%)*	*<*.*001*
*GA: week 26–27*	*4* (*21.1%)*	*2* (*14.3%)*	*2* (*40.0%)*	.*35*
Male sex	90 (57.0%)	41 (55.4%)	49 (58.3%)	.71
Antenatal steroids	138 (87.3%)	66 (89.2%)	72 (85.7%)	.76
Incomplete course	31 (19.6%)	15 (20.3%)	16 (19.0%)	.90
Complete course	107 (67.7%)	51 (68.9%)	56 (66.7%)	
PROM	57 (36.1%)	51 (68.9%)	56 (66.7%)	.98
C-section	145 (91.8%)	64 (86.5%)	81 (96.4%)	.07
BPD	45 (28.5%)	14 (18.9%)	31 (36.9%)	.025
NEC (surgical)	16 (10.1%)	5 (6.8%)	11 (13.1%)	.15
Severe IVH (Grades 3–4)	18 (11.4%)	9 (12.2%)	9 (10.7%)	.48
Severe ROP (Grad 3–4)	13 (8.2%)	4 (5.4%)	9 (10.7%)	.30
Survival free of major complications	82 (51.9%)	41 (55.4%)	41 (48.4%)	.53
SGA	19 (12.0%)	6 (8.1%)	13 (15.5%)	.16

## Discussion

The main result of this population-based, state-wide register study is a very low overall mortality rate *of 5%*. Furthermore, mortality was nearly halved over time in favor of the most immature infants below 28 weeks of GA. The low mortality rate is remarkable compared with recent nationwide Austrian (4) and Swiss (17) studies, with mortality rates of 8.4% from all live births and 9.9% of all infants admitted to the NICU, respectively. Mortality is also lower when compared to former national data from 1999 to 2001 (18). Our results are also comparable with international data from eight countries, which show an overall mortality rate of 10%, ranging from 5% in Japan to 17% in Spain (19). This study investigates infants over a similar time frame with the same neonatal demographic characteristics, mainly the same birth weight, gestational age, sex, rate of completed antenatal steroids, C-sections, and rate of multiple births like the studies cited above. In this study, we also report more than simple outcome parameters, such as mortality and morbidities, but also show a fair incidence of survival free of major complications of 73%, which is one of the most valuable concern in counseling parents.

Second, our short-term morbidity rates do not significantly differ from national data (4), despite a slightly higher incidence with an increasing change over time for BPD, whereas other morbidities showed non-significant changes over time. For BPD, countries with higher mortality rates show relatively low rates and vice versa (19, 20) which underlines our finding. National analyses in Austria (4, 18), Switzerland (17) and Germany (21) show BPD rates of 10%, 9.5% and 19.2%, respectively, international data ranges from 15% for Israel and Spain to 32% for the United Kingdom (19) and is considered a critical parameter for long-term outcomes and early death (8, 11, 22). A recent review (23) states that use of the NIH 2018 criteria identified a greater proportion of patients with BPD than did the NIH 2001 definitions (8). Numerous studies (9–11) offer approaches to factors influencing BDP, like sustained inflation, mechanical ventilation vs. CPAP, less invasive surfactant application, oxygen saturation limits or even nutrition, but incidences and treatment do not show significant changes over time. In our study, we find a decrease in the administration of antenatal steroids over time, and interpret this as an additional potential cause of our rising BPD rates. This decrease does not correspond to an expected negative impact on the IVH rate (24, 25) which might be due to the fact of the too small sample size or that other parameters may outweigh this effect e.g., hemodynamic management. Furthermore, we participated in two studies [NIPPV (9) and SAIL stud y^10^], in which BPD is diagnosed using an oxygen reduction test in all infants treated with any respiratory support (mechanical ventilation, nCPAP, nasal cannula) regardless of FiO_2_ to maintain an oxygen saturation of ≥90%. In our opinion, this definition (13), improved survival over time, an oxygen saturation target of 90%, and greater awareness may have led to more infants being eligible for this test and subsequent BPD diagnosis, especially in the *ELGAN*.

Third, the ELGAN show encouraging results, with a low and significantly decreasing mortality over time and a fair rate of survival free of major complications. However, when comparing P1–P2 we see doublings in all morbidities except IVH. We attribute this result to the decreasing mortality and the composition of the group. In P2, we see distinctly more infants younger than 25 weeks of gestational age.

In addition, our data for other short-term morbidities, severe NEC, ROP and IVH show favorable results with moderate incidences which do not change over time and can be compared to (inter)national results (4, 17, 19, 21). Caution is required in interpreting these data due to variations in case of definition (e.g., for NEC) or missing data [e.g., ROP (17, 21)].

In general, it is difficult to compare our results because of absent other national data (1, 4), different outcome parameters reported, time periods observed, and inclusion of infants in this age group. Based on the small sample size only a descriptive analysis is adequate. However, our data reflect the efforts in advancing delivery room management (9, 10, 26), but also show possibilities for improvement (26–28).

### Strength and limitations

Our study has some limitations. According to our register (ÖFGOR), all stillbirths and delivery room (DR) deaths are excluded which certainly influence the results, namely survival by underestimate of the actual mortality rate. When introducing the register in 2012 (5) we decided on a minimal dataset which did not include this parameter—as well as periventricular leucomalacia (PVL) and sepsis—because entering data is voluntary and not funded (2, 4, 5). Retrospectively, delivery room death can not be determined because live-born infants below 32 weeks of GA are not registered in national (29) or regional data sets (7). However, other studies (30, 31) also exclude DR deaths and a recent meta-analysis of 65 studies shows that this refers mainly to the ELGAN at the limit of viability (30): for infants of 25 weeks GA and above the percentage of survival of all live births and survival of infants admitted at the NICU does not show any notable differences. On the other hand, infants, where therapy was withheld after admission in the further course to the NICU, were also included in our death rates (4).

We did not adjust for risk factors because of the limited sample size. However, analyses conducted by ÖFGOR show that statistical risk predictors for death or adverse short-term outcome are not only low GA and low birth weight, but also missing or incomplete course of antenatal steroids, male sex, and multiple births (4).

To reduce the bias of different inclusion criteria, time periods, or countries with different cultures and health care systems (19, 32, 33) we describe our outcome with respect to national (4, 18), as well as to our neighboring countries Switzerland (17) and Germany (16, 21) and international data with similar timeframes and health care systems (19, 32–34). The long time period of our study may contribute to bias because patient care has changed. Certainly, the strategy in ventilatory support has undergone the biggest changes by new non-invasive measures like CPAP, NIPPV, less invasive surfactant application (LISA), or higher rates in *non-invasive ventilation*. Others remain constant like human milk feeding, or no probiotic supplementation. However, our study was not designed to quantify these effects and the number of included patients is far too limited to give any evidence. Of concern is the higher NEC rate in the ELGAN, when compared to others (16), which lead to several measures like an early implementation of human milk-based nutritional products in the latter time period of the study.

Finally, this study investigated outcome data at discharge but did not define long-term outcomes which are of great concern for patients, clinicians, and families, and affect the quality of life (12, 35).

The strength of this study is its prospective, population-based design over a long period of 14 years in a defined area. Considering the fact that data entry was based on voluntariness and no funding was available for individual centres (4), the inclusion rate seems adequate and is comparable to that of other studies: missing data in other population studies ranged from 3% in the Israel Neonatal Network to up to more than 40% in the Neonatal Research Network of Japan (19).

This is the first and only investigation of short-term outcomes in a regional setting in Austria. In this manuscript, we disclose our data and results to the public concerning benchmarks for very preterm infants and patient volumes which are strongly and repetitively discussed in health and politics (36, 37): some might argue that our group might not be representative. However, in our register, the mean birth weight, GA, sex or rate of completed antenatal steroids are the same as in the national studies for Austria and Switzerland (4, 17). In addition, we have a low rate of missing values, which reflects a good adherence to the register. Therefore, we are convinced that our cohort and subsequently our results are comparable to nationwide as well as international data.

Others might state that our patient volume is too low and the outcome might be better in a larger center (36–42). The effect of case load may be underestimated, if the results are not adjusted for the systematic referral of infants to the larger tertiary centers (40) which is not the case in our population. A recommended minimum provider volume does not exist (43) in Austria (3) nor in Germany (36, 42). The benchmark of a minimum of 30 preterm infants per year with <1,250 g birth weight was suspended by the Federal Social Court in 2015 in Germany (36) pending a final decision (42). Regionalization policies of neonatal care has been endorsed to improve neonatal outcome and geography plays a major role in how the neonatal care networks are set up and function (43). Although significant investments in infrastructure and other nationwide improvements were made in the European Union, Portugal is a country to demonstrate a significant decline in neonatal mortality coincident with closing smaller centers arguing strongly for the effect of regionalization on this outcome (41). While the positive effects of volume seem clear, the optimal threshold for discrimination low-volume from high-volume centers remains unclear (38, 41, 42). A study from Switzerland demonstrated that center variability in survival rates cannot be explained by baseline demographics or center case load (44). Providing slightly above 35 very preterm infants per year, we demonstrate one of the lowest mortality rates published in equal cohorts over an equal timeframe. Our results can be partially explained by the fact that all hospitals in our state are affiliated within a formal network with the same standard operating procedures, such as guidelines for antenatal maternal transfers or newborn resuscitation algorithms and the low rate of severe IVH may also reflect this good standard of care (36). Another explanation might be the mandatory regular attendance at simulation training programs for every team member and participation in the Video Apgar (22–26) and other trials (9, 10), which improved our DR management. Participating in such clinical studies means that we transparently expose our efforts to critical discussion within our institution and at other centers which may lead to standardization and improvement of our work.

### Further implications

Reducing morbidities among vulnerable infants remains a challenge for clinicians and investigators. Participation in (inter)national quality activities, multicenter registers, and clinical trials improves the quality of care and is increasingly considered an index of quality assurance. Precise data collection is an important component of guiding strategies for better outcomes. However, neither a continuous reduction in the viability limit nor an increase in survival rates, but rather the quality of life, should be the most important parameter in perinatal medicine.

## Conclusion

In this prospective, population-based, state-wide study, we show that the overall mortality in our cohort is low and decreases over time, mainly in the *ELGAN* born before 28 weeks of GA. Short-term morbidities and survival free of major complications do not differ as compared to nationwide Austrian and international data in a similar age group, despite lower patient numbers. The limited size does not permit any risk adjustment and so it is not possible to deviate how much of the very good outcome are due to patient characteristics. Participation in international trials is essential for improving areas of difficulty. Respiratory support strategies, hemodynamic management, and antenatal care must be targeted to improve BPD and NEC rates in our population.

## Data Availability

The original contributions presented in the study are included in the article/Supplementary Material, further inquiries can be directed to the corresponding author.
